# A Cross-Sectional Study on Central Sensitization and Autonomic Changes in Fibromyalgia

**DOI:** 10.3389/fnins.2020.00788

**Published:** 2020-08-04

**Authors:** Sandipan Hazra, Srikumar Venkataraman, Gita Handa, S. L. Yadav, Sanjay Wadhwa, U. Singh, K. P. Kochhar, K. K. Deepak, Kaushik Sarkar

**Affiliations:** ^1^Department of Physical Medicine and Rehabilitation, All India Institute of Medical Sciences, Jodhpur, India; ^2^Department of Physical Medicine and Rehabilitation, All India Institute of Medical Sciences, New Delhi, India; ^3^Department of Physiology, All India Institute of Medical Sciences, New Delhi, India; ^4^Department of Electronics and Communication Engineering, Narula Institute of Technology, Kolkata, India

**Keywords:** autonomic dysfunction, cold pressor test, cortical sensitization, deep breathing test, functional near-infrared spectroscopy, heart rate variability

## Abstract

Fibromyalgia is a multi-symptomatic disorder characterized by generalized pain. The pathophysiology of fibromyalgia is supposedly an interplay between central nervous system hyper-responsiveness, autonomic dysfunction, and peripheral pain. In this cross-sectional study, the objective was to assess central sensitization and autonomic activity in patients with fibromyalgia compared with control. Fifty adults diagnosed with fibromyalgia by the modified American College of Rheumatology 2010 criteria and an equal number of age- and sex-matched controls participated in the study in an urban tertiary care hospital. Central sensitization was assessed by history and by evidence of increased prefrontal cortical activity as measured by cortical oxygenation using functional near-infrared spectroscopy. Autonomic activity was assessed by heart rate variability, electrodermal activity, and deep breathing test in three physiological states: rest, sympathetic stress (cold pressor test), and deep breathing. Mann–Whitney *U*-test, paired *t*-test, Wilcoxon test, and Friedman test with Bonferroni *a priori* were used to analyze the data. Cortical activity was significantly higher in the fibromyalgia group than control. There was no significant difference in autonomic activity between the fibromyalgia and control groups. In the fibromyalgia group, variable degrees of sympathetic hyperactivity and normal parasympathetic activity were observed. Central sensitization may be playing a primary role in the pathophysiology of generalized pain in fibromyalgia.

## Introduction

Fibromyalgia (FM) is a multi-symptomatic disorder with a varying degree of generalized pain. It is considered to be the second most common “rheumatic” disorder, after osteoarthritis (Clauw, 2015). Currently, environmental stress and genetic predisposition are recognized as contributing factors, and the key pathophysiological mechanism is supposedly an interplay between central nervous system hyper-responsiveness, autonomic dysfunction, and muscle or peripheral pain (Staud and Rodriguez, 2006). FM is classified under the umbrella term of medically unexplained physical symptoms (MUPSs), because the etiology is unknown and the pathophysiology unclear (Kirmayer et al., 2004). Central sensitization contributes to the augmentation and amplification of pain, maybe by top-down or bottom-up processes (Häuser et al., 2015). The autonomic nervous system (ANS) dysfunction could be a cause or an effect of the disease. Meeus et al. (2013) had summarized that only moderate evidences are present regarding the autonomic changes in FM. These inconsistent changes in the ANS may be attributed to presence of different subgroups among FM patients (Giesecke et al., 2003; Plazier et al., 2015). For management, a combined pharmacological and non-pharmacological approach is used. A diverse group of medications, mostly drugs acting on the central nervous system (CNS), are used, targeting different pathophysiological pathways. EULAR has recommended both endurance and strengthening exercises, although it is not clear which of the two is the most effective (Macfarlane et al., 2017). The strength of recommendation for using medications is “weak,” regardless of the availability of level of evidence 1a (IA) level evidences (Macfarlane et al., 2017). Even ketamine, a centrally acting *N*-methyl-D-aspartate (NMDA) receptor antagonist, has been shown to reduce pain in FM (Graven-Nielsen et al., 2000). Therefore, researchers had suggested that autonomic changes may be secondary to central sensitization (Jay and Barkin, 2015; Harte et al., 2018). Currently, the studies regarding the pathophysiology of FM are insufficient and inconclusive to clearly guide the treatment approach. So there is a need to replicate studies with all the relevant parameters in the same patient population to avoid hidden subgroup confounders. In this cross-sectional study, the objective was to assess central sensitization and autonomic activity in patients with FM and to compare them with those of control.

## Materials and Methods

This cross-sectional study was conducted from February 2016 to November 2017 with the approval of the institute ethics committee (ethical approval no. IECPG-48/27.11.2015, RT-24/27.01.2016) and with the informed consent of the participants. Throughout the research work, clinical and lab assessments and data collection, management, and analysis strictly conformed to the World Medical Association Declaration of Helsinki for the ethical principles for medical research involving human subjects.

Patients with generalized body pain attending the outpatient department were screened to diagnose FM by using the 2010 American College of Rheumatology (ACR) criteria (Wolfe et al., 2010). Patients with psychiatric disorders (major depression, post-traumatic stress disorder, anxiety disorder, etc.); regional pain syndromes; hypothyroidism; any major systemic infection or illness; any known conditions having an effect on the ANS (diabetes mellitus, etc.); and any known disorder of cerebral vascular system, connective tissue (Raynaud’s phenomenon, etc.), and peripheral nerve; and those not willing to participate were excluded from the study. Fifty adults satisfying the inclusion and exclusion criteria and an equal number of age- and sex-matched controls were enrolled in the study. At baseline, a thorough clinical examination including Depression Anxiety and Stress scale (DASS-21) assessment and blood analyses including complete blood count with erythrocyte sedimentation rate (ESR), C-reactive protein (CRP), random blood sugar, liver and renal function tests, thyroid-stimulating hormone (TSH), and serum vitamin D assay were performed. Those with vitamin D deficiency were treated as per the endocrine society clinical practice guideline before enrollment into the study (Holick et al., 2011). The burden of pain was assessed before and after cold pressor test (CPT) using visual analog scale (VAS) from 0 to 10, where “0” indicates no pain and “10” indicates the worst imaginable pain.

### Assessment of Central Sensitization

**FIGURE 1:**
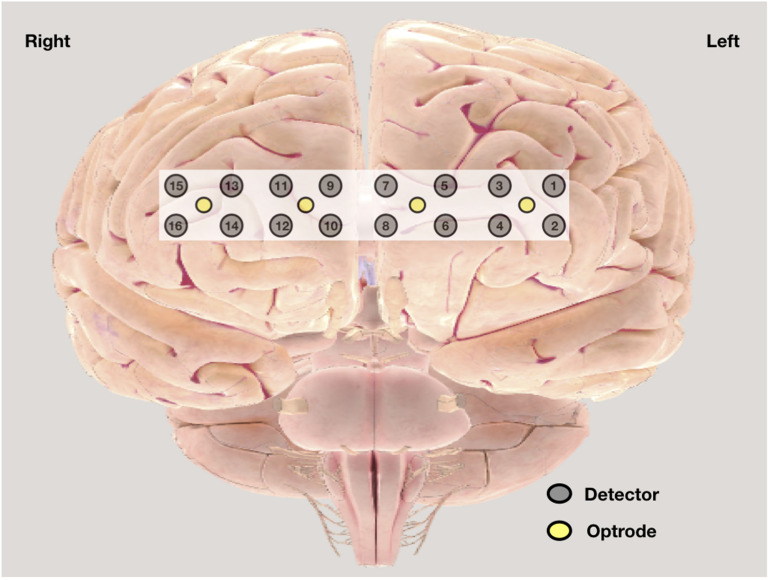
Graphic position of optrode and detectors in functional near-infrared spectroscopy (fNIRS) band.

Central sensitization was assessed by history and by evidence of cortical activation as measured by non-invasive functional near-infrared spectroscopy (fNIRS) (Woolf, 2011; Fillingim et al., 2016). In history, burden of pain, pain intensity, and affect were assessed by VAS pain scale; duration of pain was noted; and bodily distribution was assessed by widespread pain index (WPI) in ACR 2010 FM diagnostic criteria. fNIRS 300B (BIOPAC), a wearable continuous fNIRS system with four optrodes and 16 detectors (Figure 1) with 2.5 cm optrode and detector separation and approximately 1.25 cm penetration depth, was used to assess the changes in cortical activation from Brodmann areas 9, 10, 45, and 46 (Ayaz et al., 2011, 2013). The sensor has a temporal resolution of 500 ms per scan. Based on previous studies, this prefrontal region is involved in the processing of pain (Apkarian et al., 2005) and is affected in FM (Cagnie et al., 2014). Changes in oxy-hemoglobin concentration were measured during rest and during CPT and were compared with those of control. Previous studies had shown that changes in oxy-hemoglobin concentration are the most sensitive indicator of regional cerebral blood flow, which indicates regional cortical activation (Hoshi et al., 2001; Üçeyler et al., 2015). We have also documented the time taken to reach at peak oxy-hemoglobin concentration during CPT. The rectangular strap with fNIRS sensors was strapped onto the forehead just above eyebrows, with the central sensor aligned along the vertical axis of the nose and with right foot immersed in ice-cold water. The recording was analyzed with fnirSoft (BIOPAC software) for motion artifact correction and Matlab R2016a version by MathWorks, Inc. for further analysis. Time series was used for the analysis of fNIRS. In the time series, the peak amplitude was compared. An activity was analyzed only if its intensity was between 60 and 100% of maximum, that is, 100% of the maximum peak amplitude observed. In this present study, this approach was adopted to exclude doubtful recordings of activation.

### Assessment of Autonomic Function

The tests were performed in the morning (9:30–11:30 a.m.). The lab room was maintained at 22–24°C and devoid of bright colors, sounds, or bright light. Subjects were instructed to fast for at least 2 h prior to testing and not to consume coffee, nicotine, or alcohol for 24 h before the testing. It was ensured that drugs known to affect cardiac autonomic functions like anticholinergics (including antidepressants, antihistamines, and over-the-counter cough and cold medications), 9α fludrocortisone, diuretics, and sympathomimetic (α and β agonists), and para-sympathomimetic agents were stopped after consultation with the prescribing physician for 2 days before testing. All participants were instructed to wear loose and comfortable clothing.

The ANS was assessed by heart rate (HR) variability (HRV) from electrocardiography (ECG) lead II data, electrodermal activity (EDA), and deep breathing test (DBT) using BIOPAC BioNomadix MP 150 (Mathias, 2003). This was recorded during rest and at two controlled physiological stress conditions, that is, three cycles of CPT and 5 min of deep breathing (Table 1). During the test period of 5 min of rest, the participants were instructed to close the eyes and avoid talking, sleeping, coughing, and moving hands, legs, and body. CPT (Hardy et al., 1948) was used to inflict pain, which also activates the sympathetic nervous system (Keselbrener and Akselrod, 1998). In this test, the right foot was first immersed up to the ankle for 2 min in a plastic tub containing water at room temperature. Then, three serial trials were performed with ice-cold water, each trial lasting a minute and alternating with 2 min room temperature water immersion (Barati et al., 2013). During the DBT, physiological parameters were recorded when the subjects performed smooth, slow, and deep breathing at the rate of six cycles per minute. For each cycle, the inspiration is done for 5 s and expiration for 5 s. DBT parameters, that is, expiration:inspiration (E:I) ratio and delta HR were calculated from the parameters recorded (Piha, 1991). E:I ratio is the ratio of the longest R–R interval and shortest R–R interval, and delta HR is the difference between the maximal and minimal HR during inspiration and expiration, respectively. All the parameters were recorded simultaneously by fnirSoft (BIOPAC software) and BIOPAC BioNomadix MP 150.

**TABLE 1:** Physiological tests done during different conditions in the methodology.

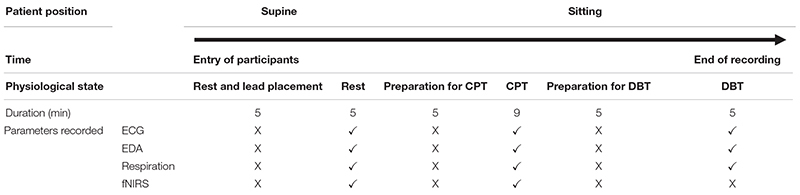

**CPT, cold pressor test; DBT, deep breathing test; EDA, electrodermal activity; fNIRS, functional near-infrared spectroscopy.**

Short-term analysis of HRV was done using a 5 min recording from lead II ECG with the participants lying supine during and after a 10 min rest period (Malik, 1996). Total power (ms^2^), low frequency (LF; ms^2^ and nu), high frequency (HF; ms^2^ and nu), and LF/HF ratio and root mean of squared successive RR intervals (RMSSD) (ms) were compared between the two groups. The artifact correction was done by manual inspection. For frequency domain methods, fast Fourier transform (FFT) using Kubios HRV Standard (version 3.0.2) was done. In this software, the HRV spectrum is calculated by FFT-based Welch’s periodogram method. EDA was ascertained using standard technique (active electrode on the palm and reference on the dorsum of hand). Baseline tonic EDA (amplitude in microsiemens) was compared between the two groups.

All the data were measured for normality by Shapiro–Wilk test. Normally distributed data were expressed as mean ± standard deviation (SD), and non-normally distributed data were expressed as median and interquartile ranges values. Mann–Whitney *U-*test was used for comparison between the FM and control groups. Wilcoxon and Friedman tests with Bonferroni *a priori* were used for comparison of frequency domain HRV data between rest and at two controlled physiological stress conditions, that is, cold CPT and 5 min of deep breathing. For comparison of categorical variable (sex), the chi-square test was used. For comparison of paired parametric data [(VAS recorded before CPT) and maximum VAS], paired *t*-test was used. *p* ≤ 0.05 was considered statistically significant with a confidence interval of 95%. For *a posteriori* power calculation, we assessed oxy-hemoglobin concentration of detector 5 between cases and control during CPT and found power of 94.35%. An IBM Statistical Package for the Social Sciences (SPSS Inc., Chicago, IL, United States) (version 23.0) was used for analysis.

## Results

Fifty adults diagnosed with FM by modified ACR 2010 criteria and an equal number of age- and sex-matched controls participated in the study. The demographic variables were comparable between the two groups. The mean age of the FM group was 38.88 ± 10.52 years and of the control group was 37.78 ± 8.56 years. Females were more in both the groups, 42:8 in the FM group and 40:10 in the control. The mean duration of FM was 42.8 ± 37.1 months (Table 2). No abnormalities were detected in the baseline blood tests. The mean WPI was 12.5 ± 3.7, and the Symptom Severity Score (SSS) was 7.0 ± 1.4, assessed with the ACR 2010 criteria. The burden of pain during rest was 6.3 ± 1.5 (VAS). The maximum pain intensity during CPT was 8.3 ± 2.3 (VAS), and this was statistically significant compared with pain at rest in the FM group (*p* < 0.05). In the control group, maximum pain intensity during CPT was 5.9 ± 2.8 and was statistically significant when compared with that of patients (*p* < 0.05). In the FM group, the mean DASS-21 depression and stress scores were 8.8 ± 5.3 and 13.6 ± 5.9, respectively (normal to mild), and anxiety was 11 ± 5.9 (moderate). There is no significant correlation between depression, stress, anxiety, and HRV frequency domain parameters (Supplementary Figure 1).

**TABLE 2:** Demographic parameters and general clinical examinations between two study groups fibromyalgia patients and controls.

Variables		FM group (*n* = 50)	Control group (*n* = 50)
Age		38.88 ± 10.52	37.78 ± 8.56
Sex	Male	8	10
	Female	42	40
Weight		62.79 ± 6.46	59.34 ± 5.32
Systolic blood pressure (mmHg)		121.66 ± 9.36	122.5 ± 14.03
Diastolic blood pressure (mmHg)		79 ± 7.20	76.9 ± 7.75
Heart rate (bpm)	Rest	84.64 ± 11.72*	80.57 ± 8.41*
	CPT	85.85 ± 11.80	84.48 ± 9.9
	DBT	88 ± 14.93	87.78 ± 10.71

Data are expressed as mean ± SD. Statistics: Mann–Whitney U-test for all parameters except sex; for sex, chi-square test. FM, fibromyalgia; CPT, cold pressor test; DBT, deep breathing test. * p < 0.05.

### Central Sensitization

**FIGURE 2:**
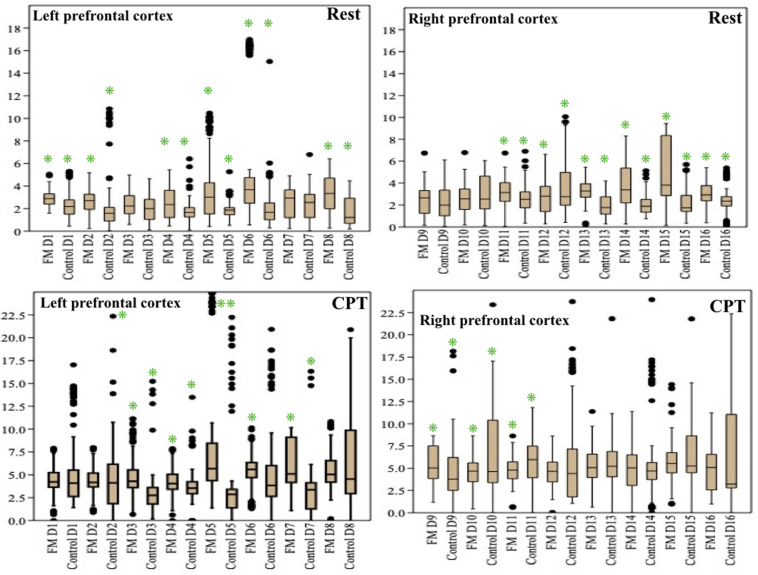
Box-and-whisker plot for peak oxy-hemoglobin concentration (μM) recorded by functional near-infrared spectroscopy (fNIRS) detectors in the prefrontal cortex during rest and cold pressor test in fibromyalgia patients and control groups. Data are expressed as median with interquartile range. Statistics: Mann–Whitney *U*-test. *p < 0.05. CPT, cold pressor test; D, fNIRS detectors in the prefrontal cortex.

**FIGURE 3:**
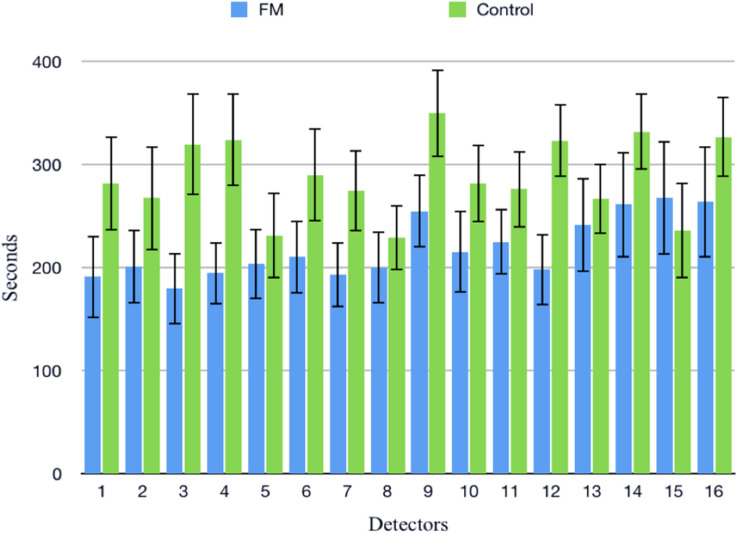
Time taken to reach at peak oxy-hemoglobin concentration (μM) in patients and control during cold pressor test in fibromyalgia patients and control groups. Data are expressed as mean and standard error; detectors: functional near-infrared spectroscopy (fNIRS) detectors in the prefrontal cortex.

The change in the pain perception in the FM group was evidenced by a significant change in VAS at rest and during CPT. The mean WPI was 12.5 ± 3.7 as described earlier. fNIRS recordings showed an increase in oxy-hemoglobin concentration at the prefrontal cortex in the patient group at rest and during CPT (Figure 2 and Supplementary Table 3). During CPT, the oxygenation was more in the left prefrontal cortex than the right in both the groups (Table 3). The time taken to reach peak oxy-hemoglobin concentration was shorter in the patient group (Figure 3).

**TABLE 3:** *p*-values representing the level of statistical significance of the difference of mean oxy-hemoglobin concentration between the corresponding pair of fNIRS detectors on the scalp in two study groups, fibromyalgia patients and controls.

Interhemispheric comparison	FM group (*n* = 50)	Control group (*n* = 50)
1 vs. 15	< 0.01*	< 0.01*
2 vs. 16	0.05*	0.78
3 vs. 13	0.01*	< 0.01*
4 vs. 14	< 0.01*	0.05
5 vs. 11	< 0.01*	< 0.01*
6 vs. 12	< 0.01*	0.54
7 vs. 9	0.96	0.03*
8 vs. 10	< 0.01*	0.54

Data are expressed as p values of comparison of oxy-hemoglobin concentration between two corresponding pair of fNIRS detectors (Figure 1) during cold pressor test by Mann–Whitney U-test. Statistics: Mann–Whitney U-test. FM, fibromyalgia; fNIRS, functional near-infrared spectroscopy. * p < 0.05.

### Autonomic Activity

**FIGURE 4:**
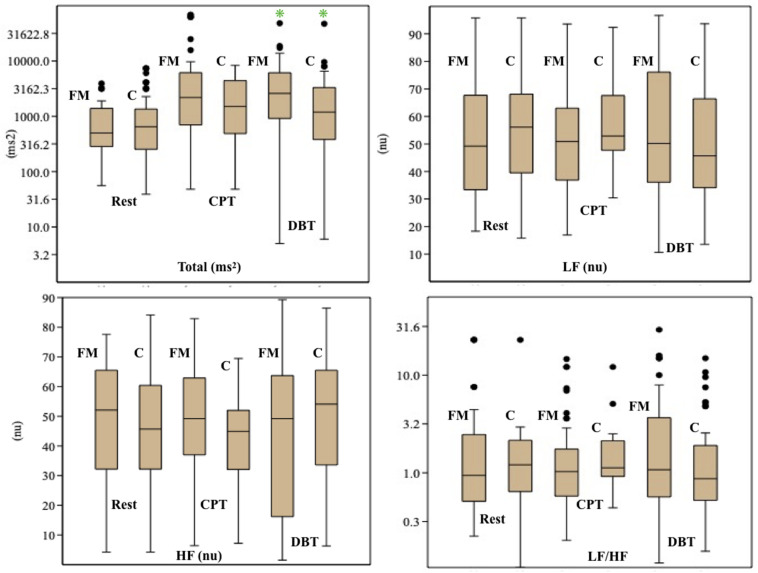
Box-and-whisker plot for comparison of frequency domain parameters of heart rate variability among fibromyalgia and control groups during rest, CPT, and DBT. Data are expressed as median with interquartile range. Statistics: Mann–Whitney *U*-test. C, control group; CPT, cold pressor test; DBT, deep breathing test; FM, fibromyalgia group; HF, high frequency; LF, low frequency; n.u., normalized unit; Total, total power. *p < 0.05.

**FIGURE 5:**
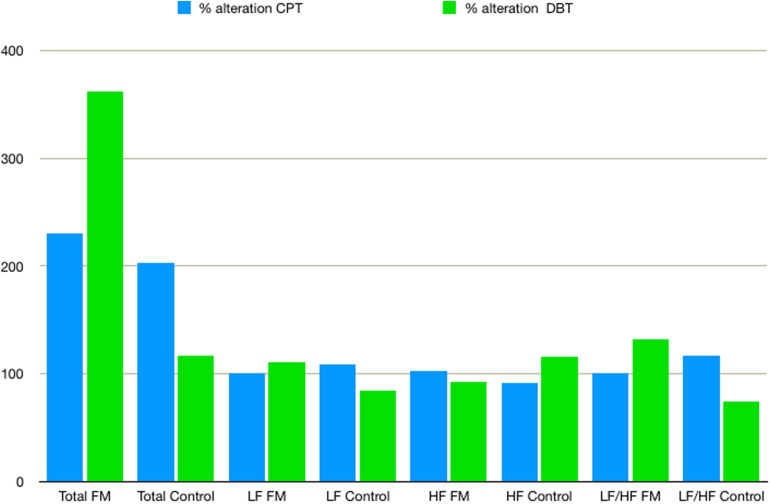
The % alteration of frequency domain parameters of heart rate variability with respect to rest of respective parameter and study group. Data are expressed as % alteration of respective parameters and study group in comparison with rest. Control, control group; CPT, cold pressor test; DBT, deep breathing test; FM, fibromyalgia group; HF, high frequency; LF, low frequency; Total, total power.

Mean HR (Table 2) was higher in the FM group than control. In both the groups, HR was increased during CPT and DBT than at rest (Table 2). At rest, total HRV was less in the FM group (i.e., total in Figure 4 and Supplementary Table 1). During CPT [% alteration compared with that of rest of respective group 230.6% (FM) vs. 202.8% (control)] and DBT [% alteration compared with that of the rest of the respective group 361.7% (FM) vs. 116.9% (control)] total power (i.e., HRV) increased more significantly in the FM group (Figures 4, 5 and Supplementary Tables 1, 2). LF (nu) was more at rest in the control group (Figure 4 and Supplementary Table 1). LF (nu) was increased with respect to rest in patients during DBT [% alteration compared with that of the rest of the FM group 100.5 (CPT)% vs. 110.5% (DBT)] and during CPT in control [% alteration compared with that of rest of the control group 108.5% (CPT) vs. 84.3% (DBT)] (Figures 4, 5 and Supplementary Tables 1, 2). HF (nu) was more in the FM group at rest compared with control (Figure 4 and Supplementary Table 1). HF (nu) component was increased with respect to rest in control during DBT and decreased during CPT [% alteration compared with the rest of the FM group 91.4% (CPT) vs. 115.6% (DBT)]. In the FM group, HF (nu) was increased during CPT and was decreased during DBT with respect to rest [% alteration compared with that of the rest of the control 102.6% (CPT) vs. 93.1% (DBT)] (Figures 4, 5 and Supplementary Tables 1, 2). RMSSD was more in the FM group than control [29.30 (17.50–60.60) vs. 28.65 (20.10–58.85] (*p* > 0.5) at rest. Parasympathetic tone as evidenced by RMSSD increases in both patients and controls during CPT [65.65 (21.50–121.70) vs. 63.40 (25.38–154.43)] (*p* > 0.5) and DBT [57.85 (22.98–100.30) vs. 44.85 (33.10–101.15)] (*p* > 0.5). LF/HF ratio was more in the control group during rest (Figure 4 and Supplementary Table 1). The increase in LF/HF ratio in the patient group was more during DBT with respect to rest [% alteration compared with the rest of the FM group 100.6% (CPT) vs. 132.2% (DBT)] (Figures 4, 5 and Supplementary Tables 1, 2). LF/HF ratio in control was increased more during CPT with respect to rest and was decreased during DBT with respect to rest [% alteration compared with the rest of control 117.2% (CPT) vs. 74.1% (DBT)] (Figures 4, 5 and Supplementary Tables 1, 2). During DBT, E:I ratio and delta HR were higher in the FM group than control but within normal limits in both (Table 4). In the FM group, tonic EDA amplitude was more at rest and CPT and the least during DBT (Table 5 and Supplementary Figure 2), whereas in the control group, it was more during DBT.

**TABLE 4:** Comparison of deep breathing test parameters between two study groups, fibromyalgia patients and control.

Variables	FM group (*n* = 50)	Control group (*n* = 50)
E:I ratio	1.56 (1.32–1.59)	1.48 (1.38–1.84)
Delta HR	32.58 (22.80–42.83)	31.11 (27.72–46.76)

**Data are expressed as median with interquartile range in parentheses. Statistics: Mann–Whitney U-test. FM, fibromyalgia; E:I, expiration:inspiration ratio; HR, heart rate; bpm, beats per minute.**

**TABLE 5:** Comparison of tonic electro-dermal amplitude (microsiemens) during rest, cold pressor test, and deep breathing test between two study groups, fibromyalgia patients and control.

Physiological state	FM group (*n* = 50)	Control group (*n* = 50)
Rest	0.005 (−0.919 to −0.045)*	−0.292 (−0.379 to 0.028)*
CPT	0.002 (−1.479 to −0.088)*	−0.335 (−1.710 to 0.096)*
DBT	−0.002 (−0.950 to −0.081)	−0.274 (−1.1465 to 0.101)

Data are expressed as median with interquartile range in parentheses. Statistics: Mann–Whitney U-test. bpm, beats per minute; CPT, cold pressor test; DBT, deep breathing test. * p < 0.05.

## Discussion

In this study, generalized widespread moderate body pain at rest and a significant increase in pain intensity during CPT were observed in the FM group when compared with that of control. During CPT and at rest, increased oxy-hemoglobin concentration was observed in the prefrontal cortex in the FM group, and this increase in regional cortical blood flow (Hoshi et al., 2001) objectively demonstrates an alteration in cortical activity in the FM group. Changes in central nervous system activity along with hyperalgesia, moderate pain burden at rest, and widespread bodily pain in FM patients probably point toward central sensitization (Woolf, 2011; Fillingim et al., 2016). Higher oxy-hemoglobin level at rest and in response to pain in the prefrontal cortex of FM patients also indicated altered central nervous system connections and processing (Cagnie et al., 2014; Flodin et al., 2014; Üçeyler et al., 2015). Owing to immersion of right foot in cold water (pain stimulus), there was an increase in oxy-hemoglobin concentration at the contralateral prefrontal cortex (left) (Table 3).

During CPT, an increase in HR was observed in both the groups owing to sympathetic overdrive. During DBT, an increase in HR was observed in both the groups owing to interruption of cardiac vagal discharge and the rhythmic activity of the cardiac sympathetic system (Wright et al., 1982).

Total power in HRV increased significantly during CPT and DBT. The increase in% alteration was more in the FM group. Kulshreshtha et al. (2012) and Zamunér et al. (2016) also described similar findings. However, the rate of increment of total power during DBT was different from that of Zamunér et al. The% alteration of total power compared with the rest of in the FM group was 361.7% in our study vs. 479.3% in the study by Zamunér et al. and in the control group of 116.9% in our study vs. 531.7% in the study by Zamunér et al. This could be due to the difference in the increase of LF and HF, differences in mean age, and duration of ECG recording (4 vs. 5 min). This significant increase in total power in HRV during CPT and DBT in patients with FM may reflect a poor feedback control as described by previous studies (Jay and Barkin, 2015). An increase in the LF component of HRV generally poorly correlated with increased sympathetic activity (Houle and Billman, 1999). Our finding of reduced the LF component at rest and a lesser amount of % alteration of LF during CPT than DBT in the patient group was supported by Kulshreshtha et al. (2012). Jay et al. also summarized that the sympathetic nervous system of FM patients responds poorly to pain (Jay and Barkin, 2015). Martínez-Lavín et al. (1998) and Cohen et al. (2000) reported increased the LF component in FM patients during rest, which is inconsistent to our finding. The decrease in the LF component at rest could be due to added vagal stimulation (Hedman et al., 1995). Blunted sympathetic response to stress like cold and pain is the cause for the decrease in the LF in the FM group (Kulshreshtha and Deepak, 2013; Jay and Barkin, 2015). An increase in the LF component was observed in the patient group during DBT. This was described in other studies. Brown et al. described an increase of the LF component during slow breathing in normal subjects (Brown et al., 1993). Patwardhan et al. (1995a, b) concluded that there was a decrease in the parasympathetic influence of cardiovascular regulation during controlled breathing, and they proposed a role of respiratory pattern generator behind this finding. Bhagat et al. (2017) proposed that a dynamic interaction by mechanical coupling, baroreflex, and central cardiovascular control between respiratory and cardiovascular system causes an increase in the LF component. The authors also correlated this with the duration of monitoring (Freeman, 2009). As mentioned earlier, the LF component probably represents the interaction of the sympathetic and parasympathetic nervous systems. The increase in the LF component may reflect an increase in parasympathetic tone during DBT (Axelrod et al., 1997; Russo et al., 2017). The HF component was more in the FM group during rest. In the FM group, HF increased during CPT and decreased during DBT [102.6% (CPT) vs. 93.1% (DBT)]. Weise et al. (1993) also described a similar finding of the increase of HF during CPT and suggested that changes in ventilation are because of the change in venous return and/or intra-thoracic pressure. On the other hand,% alteration of HF increased in the control group during DBT. The HF% increase during CPT and decrease during DBT also suggested a poor feedback control to stress in the patient group. LF/HF ratio was more in the FM group during rest. The % alteration of LF/HF ratio increased in the patient group during CPT and DBT [100.6% (CPT) vs. 132.2% (DBT)]. This indicates increasing sympathetic predominance during provocation. This is supported by previous studies (Martínez-Lavín et al., 1998; Kulshreshtha et al., 2012; Zamunér et al., 2016). Bianchi et al. (1990) supported an increase of this ratio during DBT. RMSSD, HF, and decreased LF/HF ratio indicated intact parasympathetic tone in our study in the patient group.

In DBT, E:I ratio and delta HR were above the normal cutoff in both cases and controls. This also showed an evidence of intact parasympathetic system in FM patients. The previous studies reported mixed results (Kulshreshtha et al., 2012; Zamunér et al., 2016).

Tonic EDA was reported in this study as the phasic EDA latency and amplitude lack sensitivity and specificity. The authors have consistently suggested to study tonic EDA amplitude (Mathias and Bannister, 2013). Tonic EDA was more in the FM group than control, at rest and during CPT, which was probably due to an increased sympathetic activity in patients with FM. During DBT, tonic EDA was more in the control group than the FM group, which could be due to disharmony between sympathetic and parasympathetic systems. No previous study has reported tonic EDA.

**FIGURE 6:**
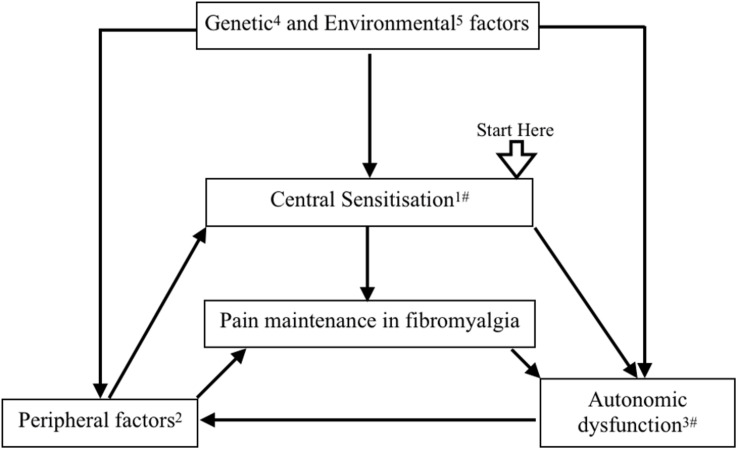
A flow diagram proposing pathophysiology of fibromyalgia. Central sensitization has been proposed as one of the key pathophysiological mechanisms of fibromyalgia. Activation of peripheral pain receptor due to muscle pathology, regional ischemia due to autonomic dysfunction, and small fiber neuropathy cause pain generation and maintenance in muscles. Genetic and epigenetic modifications provide a background of peripheral factors and central sensitization. Stress, infection, vaccination, and environmental factors have been related with peripheral, central, and autonomic changes in fibromyalgia. Central sensitization and peripheral pain influence the autonomic system. All these factors make a positive feedback loop for pain maintenance in fibromyalgia. **(1)** Yunus (2008); Woolf (2011), Häuser et al. (2015); Harte et al. (2018). (**2**) Bengtsson (2002); Kulshreshtha et al. (2012), Harte et al. (2018). (**3**) Kulshreshtha et al. (2012); Meeus et al. (2013), Jay and Barkin (2015). **(4)** Diatchenko et al. (2013); D’Agnelli et al. (2019). (5) Buskila et al. (2008); Albrecht and Rice (2016). ^#^Our study supports this mechanism.

Statistical difference in mean between the two groups was observed in only a few of the autonomic variables (Supplementary Tables 1, 2). Wide variability among the clinical clusters of FM patients could be a reason (Giesecke et al., 2003; Plazier et al., 2015). Elucidation of pathophysiological correlation among these clinical clusters could be a scope of further research. These may be the cause of the difference in the results of previous studies on autonomic variables in patients with FM (Meeus et al., 2013; Martínez-Martínez et al., 2014). In this study, increased cortical activity with probable dysautonomia was observed in FM patients. So our study supports the hypothesis proposed by Yunus et al., and Hause et al., who reported that CNS changes distort or amplify pain, which causes hypersensitivity to pain. Pain causes sympathetic over-activity, which leads to a vicious cycle of pain maintenance in FM (Yunus, 2008; Häuser et al., 2015; Harte et al., 2018; Figure 6).

Earlier, most of the studies had assessed dysautonomia in patients with FM in a single physiological state and a single parameter. In this study, multiple parameters, namely, HRV, EDA, E:I ratio, and delta HR, were observed in three different physiological states, that is, at rest and at two stress conditions. Cortical neurovascular activity was also assessed by fNIRS in the same patient population. Thus, the key pathophysiological components of FM, that is, central sensitization and dysautonomia, were assessed in this study. A study with multiple parameters may be an ideal approach to explore the pathophysiology of medically unexplained symptoms like FM. Some impediments and limitations were faced during the design of this study. ACR 2010 criteria for diagnosis of FM were used instead of the 2011 revision. Also, body mass index and physical activity of participants were not assessed. Variability of autonomic functions on posture of recording, BMI, and level of physical activity among the participants were not considered. Also, the contribution of peripheral factors like small fiber neuropathy has not been evaluated in the current study. The recommended period for DBT varies from 60 to 90 s (Piha, 1991), and the recommended period for short-term HRV analysis by ECG is 5 min (Malik, 1996). In this study, emphasis was placed on HRV analysis by ECG, and the prolonged duration of CPT and DBT could have dampened the response of ANS to stress. fNIRS, a low-cost non-invasive tool, was used for assessment of cortical neurovascular activity. Deeper structures involved in pain processing in FM were not accessible with fNIRS. Lack of 3D digitizer restricted the ability to localize cortical activity. Also, there is a need to determine the cut-off for oxy-hemoglobin concentration paradigm to exclude doubtful activations.

In this study, patients with FM patient probably had central sensitization with equivocal sympathetic hyper-reactivity and a blunted response to stress, an intact parasympathetic system. Thus, this study supports that hypothesis that generalized pain in FM is probably due to central nervous system hypersensitivity.

## Data Availability Statement

The raw data supporting the conclusions of this article will be made available by the authors, without undue reservation.

## Ethics Statement

The studies involving human participants were reviewed and approved by Institute Ethics Committee for Post Graduate Research, All India Institute of Medical Sciences, New Delhi. The patients/participants provided their written informed consent to participate in this study.

## Author Contributions

SH: designing the study, recruiting patients and controls, performing the tests, analyzing the results, and preparing the manuscript. SV: designing the study, recruiting patients and controls, analyzing the results, providing expert opinion, and preparing the manuscript. GH, SW, and US: designing the study, recruiting patients and controls, providing expert opinion, and preparing the manuscript. SY: expert opinion. KK: designing the study and expert opinion. KD: designing the study, analyzing the results, providing expert opinion and pathophysiological co-relations, and preparing the manuscript. KS: designing the software code and preparing the manuscript. All the authors have contributed in designing and conduct of study, analysis of results and preparation of manuscript.

## Conflict of Interest

The authors declare that the research was conducted in the absence of any commercial or financial relationships that could be construed as a potential conflict of interest.

## Supplementary Material

FIGURE S1 Spearman’s Correlation between frequency domain parameters (LF, HF, and LF/HF ratio), visual analog scale (11-point scale), widespread pain index (WPI) [American College of Rheumatology (ACR) fibromyalgia diagnostic criteria 2010] and Depression Anxiety and Stress scale (DASS-21). Data are expressed as r_s_ value of Spearman’s Correlation; r_s_ value of Spearman’s Correlation are gradient filled color coded bar on the left side of plot; blue color denotes positive correlation, red color denotes negative correlation, darker opacities denotes stronger correlation; C: control group; CPT: cold pressor test; DBT: deep breathing test; Depression (D) Anxiety (A) and Stress (S) scale (DASS-21); FM, fibromyalgia group; HF, high frequency; LF, low frequency; VAS, visual analog scale (11-point scale); WPI, widespread pain index [American College of Rheumatology (ACR) fibromyalgia diagnostic criteria 2010] and boxed values signify *p* < 0.05.

FIGURE S2 Box and Whisker plot for comparison of tonic electro-dermal amplitude (microsiemens) among fibromyalgia and control group during rest, CPT and DBT. Data are expressed as median with interquartile range; Statistics: Mann-Whitney *U*-test; C, control group; CPT, cold pressor test; DBT, deep breathing test; FM, fibromyalgia group; ^∗^*p* < 0.05.

TABLE S1 Comparison of frequency domain parameters of heart rate variability among fibromyalgia and control group during rest, CPT and DBT.

TABLE S2 Comparison of frequency domain parameters of heart rate variability among fibromyalgia and control group during rest, CPT and DBT by Wilcoxon and Friedman test with Bonferroni priori.

TABLE S3 Comparison of peak Oxy-hemoglobin concentration (μM) recorded by fNIRS detectors in the prefrontal cortex during rest and cold pressor test in fibromyalgia patients and control groups.

## References

[B1] Albrecht P. J. Rice F. L. (2016). Fibromyalgia syndrome pathology and environmental influences on afflictions with medically unexplained symptoms. *Rev. Environ. Health* 31 281–294. 10.1515/reveh-2015-0040 27105483

[B2] Apkarian A. V. Bushnell M. C. Treede R.-D. Zubieta J.-K. (2005). Human brain mechanisms of pain perception and regulation in health and disease. *Eur. J. Pain Lond. Engl.* 9 463–484. 10.1016/j.ejpain.2004.11.001 15979027

[B3] Axelrod F. B. Putman D. Berlin D. Rutkowski M. (1997). Electrocardiographic measures and heart rate variability in patients with familial dysautonomia. *Cardiology* 88 133–140. 10.1159/000177319 9096912

[B4] Ayaz H. Onaral B. Izzetoglu K. Shewokis P. A. McKendrick R. Parasuraman R. (2013). Continuous monitoring of brain dynamics with functional near infrared spectroscopy as a tool for neuroergonomic research: empirical examples and a technological development. *Front. Hum. Neurosci.* 7:871. 10.3389/fnhum.2013.00871 24385959 PMC3866520

[B5] Ayaz H. Shewokis P. A. Curtin A. Izzetoglu M. Izzetoglu K. Onaral B. (2011). Using MazeSuite and functional near infrared spectroscopy to study learning in spatial navigation. *J. Vis. Exp.* 8:3443. 10.3791/3443 22005455 PMC3227178

[B6] Barati Z. Shewokis P. A. Izzetoglu M. Polikar R. Mychaskiw G. Pourrezaei K. (2013). Hemodynamic response to repeated noxious cold pressor tests measured by functional near infrared spectroscopy on forehead. *Ann. Biomed. Eng.* 41 223–237. 10.1007/s10439-012-0642-0 22956158

[B7] Bengtsson A. (2002). The muscle in fibromyalgia. *Rheumatology* 41 721–724. 10.1093/rheumatology/41.7.721 12096218

[B8] Bhagat O. L. Kharya C. Jaryal A. Deepak K. K. (2017). Acute effects on cardiovascular oscillations during controlled slow yogic breathing. *Indian J. Med. Res.* 145 503–512. 10.4103/ijmr.IJMR_830_15 28862183 PMC5663165

[B9] Bianchi A. Bontempi B. Cerutti S. Gianoglio P. Comi G. Sora M. G. N. (1990). Spectral analysis of heart rate variability signal and respiration in diabetic subjects. *Med. Biol. Eng. Comput.* 28:205. 10.1007/BF02442668 2377001

[B10] Brown T. E. Beightol L. A. Koh J. Eckberg D. L. (1993). Important influence of respiration on human R-R interval power spectra is largely ignored. *J. Appl. Physiol.* 75:2310–2317. 10.1152/jappl.1993.75.5.2310 8307890

[B11] Buskila D. Atzeni F. Sarzi-Puttini P. (2008). Etiology of fibromyalgia: the possible role of infection and vaccination. *Autoimmun. Rev.* 8 41–43. 10.1016/j.autrev.2008.07.023 18706528

[B12] Cagnie B. Coppieters I. Denecker S. Six J. Danneels L. Meeus M. (2014). Central sensitization in fibromyalgia? A systematic review on structural and functional brain MRI. *Semin. Arthritis Rheum.* 44 68–75. 10.1016/j.semarthrit.2014.01.001 24508406

[B13] Clauw D. J. (2015). Fibromyalgia and related conditions. *Mayo Clin. Proc.* 90 680–692. 10.1016/j.mayocp.2015.03.014 25939940

[B14] Cohen H. Neumann L. Shore M. Amir M. Cassuto Y. Buskila D. (2000). Autonomic dysfunction in patients with fibromyalgia: application of power spectral analysis of heart rate variability. *Semin. Arthritis Rheum.* 29 217–227. 10.1016/s0049-0172(00)80010-4 10707990

[B15] D’Agnelli S. Arendt-Nielsen L. Gerra M. C. Zatorri K. Boggiani L. Baciarello M. (2019). Fibromyalgia: genetics and epigenetics insights may provide the basis for the development of diagnostic biomarkers. *Mol. Pain* 15:1744806918819944. 10.1177/1744806918819944 30486733 PMC6322092

[B16] Diatchenko L. Fillingim R. B. Smith S. B. Maixner W. (2013). The phenotypic and genetic signatures of common musculoskeletal pain conditions. *Nat. Rev. Rheumatol.* 9 340–350. 10.1038/nrrheum.2013.43 23545734 PMC3991785

[B17] Fillingim R. B. Loeser J. D. Baron R. Edwards R. R. (2016). Assessment of chronic pain: domains, methods, and mechanisms. *J. Pain Off. J. Am. Pain Soc.* 17 T10–T20. 10.1016/j.jpain.2015.08.010 27586827 PMC5010652

[B18] Flodin P. Martinsen S. Löfgren M. Bileviciute-Ljungar I. Kosek E. Fransson P. (2014). Fibromyalgia is associated with decreased connectivity between pain- and sensorimotor brain areas. *Brain Connect.* 4 587–594. 10.1089/brain.2014.0274 24998297 PMC4202907

[B19] Freeman R. L. (2009). “Noninvasive evaluation of heart rate: time and frequency domains,” in *Clinical Autonomic Disorders*, eds Low P. A. Benarroch E. E. (Philadelphia: Lippincott Williams & Wilkins), 197–209.

[B20] Giesecke T. Williams D. A. Harris R. E. Cupps T. R. Tian X. Tian T. X. (2003). Subgrouping of fibromyalgia patients on the basis of pressure-pain thresholds and psychological factors. *Arthritis Rheum.* 48 2916–2922. 10.1002/art.11272 14558098

[B21] Graven-Nielsen T. Aspegren Kendall S. Henriksson K. G. Bengtsson M. Sörensen J. Johnson A. (2000). Ketamine reduces muscle pain, temporal summation, and referred pain in fibromyalgia patients. *Pain* 85 483–491. 10.1016/s0304-3959(99)00308-5 10781923

[B22] Hardy J. D. Wolff H. G. Goodell H. (1948). Studies on pain: an investigation of some quantitative aspects of the DOL scale of pain intensity. *J. Clin. Invest.* 27 380–386. 10.1172/JCI101969 16695620 PMC438878

[B23] Harte S. E. Harris R. Clauw D. J. (2018). The neurobiology of central sensitization. *J. Appl. Biobehav. Res.* 23:e12137. 10.1111/jabr.12137

[B24] Häuser W. Ablin J. Fitzcharles M.-A. Littlejohn G. Luciano J. V. Usui C. (2015). Fibromyalgia. *Nat. Rev. Dis. Primer* 1:15022. 10.1038/nrdp.2015.22 27189527

[B25] Hedman A. E. Tahvanainen K. U. Hartikainen J. E. Hakumäki M. O. (1995). Effect of sympathetic modulation and sympatho-vagal interaction on heart rate variability in anaesthetized dogs. *Acta Physiol. Scand.* 155 205–214. 10.1111/j.1748-1716.1995.tb09965.x 8669293

[B26] Holick M. F. Binkley N. C. Bischoff-Ferrari H. A. Gordon C. M. Hanley D. A. Heaney R. P. (2011). Evaluation, treatment, and prevention of vitamin d deficiency: an endocrine society clinical practice guideline. *J. Clin. Endocrinol. Metab.* 96 1911–1930. 10.1210/jc.2011-0385 21646368

[B27] Hoshi Y. Kobayashi N. Tamura M. (2001). Interpretation of near-infrared spectroscopy signals: a study with a newly developed perfused rat brain model. *J. Appl. Physiol. Bethesda Md.* 1985 1657–1662. 10.1152/jappl.2001.90.5.1657 11299252

[B28] Houle M. S. Billman G. E. (1999). Low-frequency component of the heart rate variability spectrum: a poor marker of sympathetic activity. *Am. J. Physiol.* 276 H215–H223. 9887035 10.1152/ajpheart.1999.276.1.H215

[B29] Jay G. W. Barkin R. L. (2015). Fibromyalgia. *Dis. Mon.* 61 66–111. 10.1016/j.disamonth.2015.01.002 25769552

[B30] Keselbrener L. Akselrod S. (1998). “Autonomic responses to blockades and provocations,” in *Clinical Guide to Cardiac Autonomic Tests*, ed. Malik M. (Netherlands: Springer), 101–148.

[B31] Kirmayer L. J. Groleau D. Looper K. J. Dao M. D. (2004). Explaining medically unexplained symptoms. *Can. J. Psychiatry Rev. Can. Psychiatr.* 49 663–672. 10.1177/070674370404901003 15560312

[B32] Kulshreshtha P. Deepak K. K. (2013). Autonomic nervous system profile in fibromyalgia patients and its modulation by exercise: a mini review. *Clin. Physiol. Funct. Imaging* 33 83–91. 10.1111/cpf.12000 23383685

[B33] Kulshreshtha P. Gupta R. Yadav R. K. Bijlani R. L. Deepak K. K. (2012). A comprehensive study of autonomic dysfunction in the fibromyalgia patients. *Clin. Auton. Res.* 22 117–122. 10.1007/s10286-011-0150-6 22038566

[B34] Macfarlane G. J. Kronisch C. Dean L. E. Atzeni F. Häuser W. Fluß E. (2017). EULAR revised recommendations for the management of fibromyalgia. *Ann. Rheum. Dis.* 76 318–328. 10.1136/annrheumdis-2016-209724 27377815

[B35] Malik M. (1996). Heart rate variability. standards of measurement, physiological interpretation, and clinical use. task force of the european society of cardiology and the north american society of pacing and electrophysiology. *Eur. Heart J.* 17 354–381. 8737210

[B36] Martínez-Lavín M. Hermosillo A. G. Rosas M. Soto M.-E. (1998). Circadian studies of autonomic nervous balance in patients with fibromyalgia: a heart rate variability analysis. *Arthritis Rheum.* 41 1966–1971. 10.1002/1529-0131(199811)41:11<1966::AID-ART11<3.0.CO;2-O 9811051

[B37] Martínez-Martínez L.-A. Mora T. Vargas A. Fuentes-Iniestra M. Martínez-Lavín M. (2014). Sympathetic nervous system dysfunction in fibromyalgia, chronic fatigue syndrome, irritable bowel syndrome, and interstitial cystitis: a review of case-control studies. *J. Clin. Rheumatol. Pract. Rep. Rheum. Musculoskelet. Dis.* 20 146–150. 10.1097/RHU.0000000000000089 24662556

[B38] Mathias C. (2003). Autonomic diseases: clinical features and laboratory evaluation. *J. Neurol. Neurosurg. Psychiatry* 74 iii31–iii41. 10.1136/jnnp.74.suppl_3.iii31 12933912 PMC1765633

[B39] Mathias C. J. Low D. A. Iodice V. Bannister R. (2013). “Investigation of autonomic disorders,” in *Autonomic Failure: A Textbook of Clinical Disorders of the Autonomic Nervous System*, eds Mathias C. J. Bannister R. (Oxford: Oxford University Press), 259–289.

[B40] Meeus M. Goubert D. De Backer F. Struyf F. Hermans L. Coppieters I. (2013). Heart rate variability in patients with fibromyalgia and patients with chronic fatigue syndrome: a systematic review. *Semin. Arthritis Rheum.* 43 279–287. 10.1016/j.semarthrit.2013.03.004 23838093

[B41] Patwardhan A. R. Evans J. M. Bruce E. N. Eckberg D. L. Knapp C. F. (1995a). Voluntary control of breathing does not alter vagal modulation of heart rate. *J. Appl. Physiol.* 1985 2087–2094. 10.1152/jappl.1995.78.6.2087 7665403

[B42] Patwardhan A. R. Vallurupalli S. Evans J. M. Bruce E. N. Knapp C. F. (1995b). Override of spontaneous respiratory pattern generator reduces cardiovascular parasympathetic influence. *J. Appl. Physiol.* 1985 1048–1054. 10.1152/jappl.1995.79.3.1048 8567501

[B43] Piha S. J. (1991). Cardiovascular autonomic reflex tests: normal responses and age-related reference values. *Clin. Physiol.* 11 277–290. 10.1111/j.1475-097x.1991.tb00459.x 1893685

[B44] Plazier M. Ost J. Stassijns G. De Ridder D. Vanneste S. (2015). Pain characteristics in fibromyalgia: understanding the multiple dimensions of pain. *Clin. Rheumatol.* 34 775–783. 10.1007/s10067-014-2736-6 25048743

[B45] Russo M. A. Santarelli D. M. O’Rourke D. (2017). The physiological effects of slow breathing in the healthy human. *Breathe* 13 298–309. 10.1183/20734735.009817 29209423 PMC5709795

[B46] Staud R. Rodriguez M. E. (2006). Mechanisms of Disease: pain in fibromyalgia syndrome. *Nat. Clin. Pract. Rheumatol.* 2 90–98. 10.1038/ncprheum0091 16932662

[B47] Üçeyler N. Zeller J. Kewenig S. Kittel-Schneider S. Fallgatter A. J. Sommer C. (2015). Increased cortical activation upon painful stimulation in fibromyalgia syndrome. *BMC Neurol.* 15:210. 10.1186/s12883-015-0472-4 26486985 PMC4618366

[B48] Weise F. Laude D. Girard A. Zitoun P. Siché J.-P. Elghozi J.-L. (1993). Effects of the cold pressor test on short-term fluctuations of finger arterial blood pressure and heart rate in normal subjects. *Clin. Auton. Res.* 3 303–310. 10.1007/BF01827331 8124062

[B49] Wolfe F. Clauw D. J. Fitzcharles M.-A. Goldenberg D. L. Katz R. S. Mease P. (2010). The american college of rheumatology preliminary diagnostic criteria for fibromyalgia and measurement of symptom severity. *Arthritis Care Res.* 62 600–610. 10.1002/acr.20140 20461783

[B50] Woolf C. J. (2011). Central sensitization: implications for the diagnosis and treatment of pain. *Pain* 152 S2–S15. 10.1016/j.pain.2010.09.030 20961685 PMC3268359

[B51] Wright S. Keele C. A. Neil E. Joels N. (1982). “The heart and circulation,” in *Samson Wright’s Applied Physiology*, eds Keele C. A. Neil E. Joels N. (Oxford: Oxford University Press), 65–157.

[B52] Yunus M. B. (2008). Central sensitivity syndromes: a new paradigm and group nosology for fibromyalgia and overlapping conditions, and the related issue of disease versus illness. *Semin. Arthritis Rheum.* 37 339–352. 10.1016/j.semarthrit.2007.09.003 18191990

[B53] Zamunér A. R. Forti M. Andrade C. P. Avila M. A. da Silva E. (2016). Respiratory sinus arrhythmia and its association with pain in women with fibromyalgia syndrome. *Pain Pract. Off. J. World Inst. Pain* 16 704–711. 10.1111/papr.12321 26032241

