# Supplementary figures and images for: A Cross-Sectional Study on Central Sensitization and Autonomic Changes in Fibromyalgia
Source: Front Neurosci. 2020 Aug 4;14:788. doi: 10.3389/fnins.2020.00788 (PMC7417433; doi:10.3389/fnins.2020.00788)

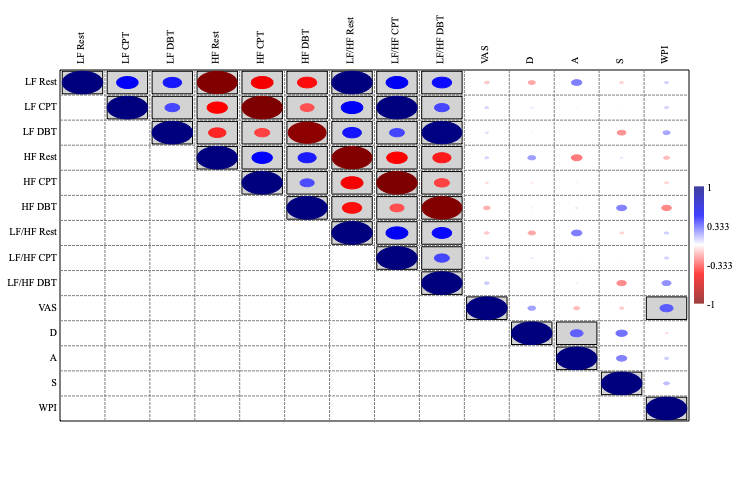

Supplement: FIGURE S1 — Spearman’s Correlation between frequency domain parameters (LF, HF, and LF/HF ratio), visual analog scale (11-point scale), widespread pain index (WPI) [American College of Rheumatology (ACR) fibromyalgia diagnostic criteria 2010] and Depression Anxiety and Stress scale (DASS-21). Data are expressed as rs value of Spearman’s Correlation; rs value of Spearman’s Correlation are gradient filled color coded bar on the left side of plot; blue color denotes positive correlation, red color denotes negative correlation, darker opacities denotes stronger correlation; C: control group; CPT: cold pressor test; DBT: deep breathing test; Depression (D) Anxiety (A) and Stress (S) scale (DASS-21); FM, fibromyalgia group; HF, high frequency; LF, low frequency; VAS, visual analog scale (11-point scale); WPI, widespread pain index [American College of Rheumatology (ACR) fibromyalgia diagnostic criteria 2010] and boxed values signify p < 0.05. [file Image_1.TIF]

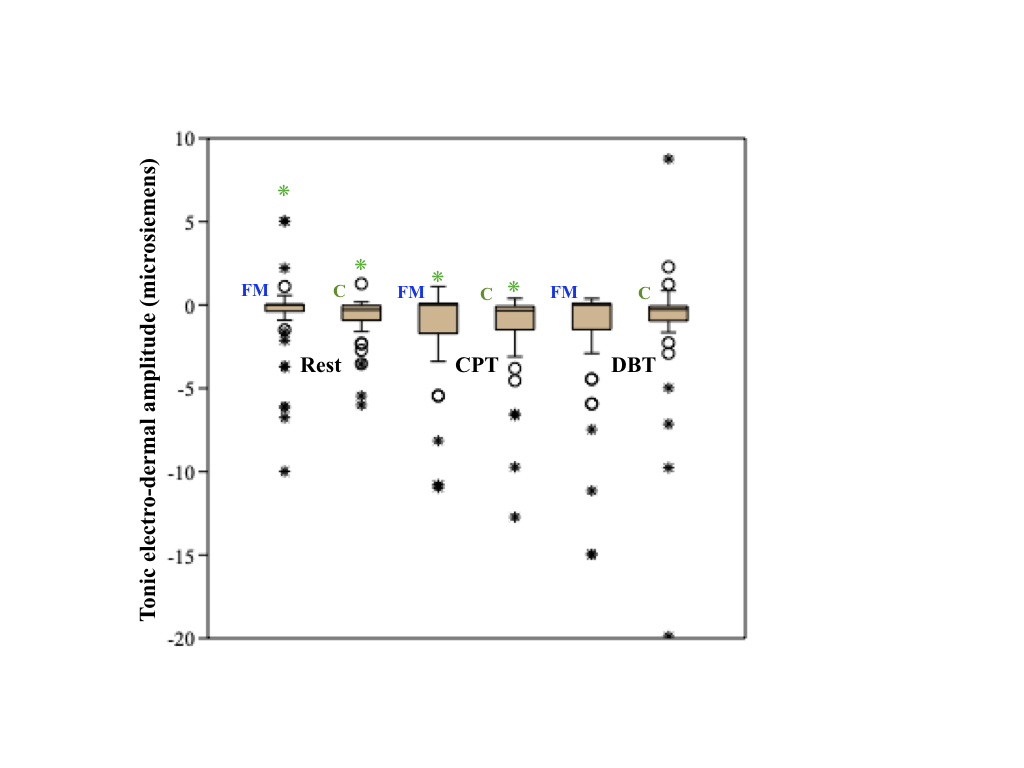

Supplement: FIGURE S2 — Box and Whisker plot for comparison of tonic electro-dermal amplitude (microsiemens) among fibromyalgia and control group during rest, CPT and DBT. Data are expressed as median with interquartile range; Statistics: Mann-Whitney U-test; C, control group; CPT, cold pressor test; DBT, deep breathing test; FM, fibromyalgia group; ∗p < 0.05. [file Image_2.TIFF]
