# Supplementary material for: A Cross-Sectional Study on Central Sensitization and Autonomic Changes in Fibromyalgia
Source: Front Neurosci. 2020 Aug 4;14:788. doi: 10.3389/fnins.2020.00788 (PMC7417433; doi:10.3389/fnins.2020.00788)
Supplement: TABLE S1 — Comparison of frequency domain parameters of heart rate variability among fibromyalgia and control group during rest, CPT and DBT. [file Data_Sheet_1.zip › Table S1.docx]

**A Cross-Sectional Study on Central Sensitization and Autonomic Changes in Fibromyalgia**

**Hazra S^1^, Venkataraman S^2^, Handa G^2^, Yadav SL^2^, Wadhwa S^2^, Singh U^2^, Kochhar KP^3^, Deepak KK^3^, Sarkar K^4^**

**Supplementary table 1 Comparison of frequency domain parameters of heart rate variability among fibromyalgia and control group during rest, CPT and DBT**

|  | **FM Group (n=50)** | **Control group (n=50)** | **p Value** |
| --- | --- | --- | --- |
| **Total Rest (ms^2^)** | 498.00 (253.00 - 1581.50) | 647.00 (282.50 - 1434.00) | 0.42 |
| **Total CPT (ms^2^)** | 2189.00 (472.75 - 4416.00) | 1488.50 (651.25 - 6167.50) | 0.22 |
| **Total DBT (ms^2^)** | 2595.50 (379.25 - 3551.25) | 1177.50 (897.00 - 6120.00) | **0.05*** |
| **LF Rest (ms^2^)** | 333.00 (107-821.25) | 269.00 (107.5 – 643.25) | 0.82 |
| **LF CPT (ms^2^)** | 941.4 (417.25-10616) | 683.50 (241.75-3652.75) | 0.15 |
| **LF DBT (ms^2^)** | 1449.5 (383.00 – 6947.00) | 345.50 (107.00-4543.00) | **0.02*** |
| **LF Rest (nu)** | 48.40(39.00 - 68.23) | 54.60 (33.40 - 70.20) | 0.86 |
| **LF CPT (nu)** | 50.55(47.70 - 67.88) | 52.90 (36.50 - 63.55) | 0.13 |
| **LF DBT (nu)** | 49.40 (34.10 - 67.50) | 45.45 (36.10 - 78.03) | 0.20 |
| **HF Rest (ms^2^)** | 255.00 (111.50 -1031.00) | 245.00 (108.50 – 847.00) | 0.76 |
| **HF CPT (ms^2^)** | 1590.00 (264.75 – 8938.00) | 459.00 (153.75 – 3382.50) | 0.10 |
| **HF DBT (ms^2^)** | 1199 (402.00 – 3155.75) | 784.00 (132.00 – 3760.00) | 0.14 |
| **HF Rest (nu)** | 51.45 (32.13 - 60.90) | 44.80 (29.68 - 65.50) | 0.90 |
| **HF CPT (nu)** | 49.10 (31.90 - 52.00) | 43.85 (36.43 - 63.20) | 0.11 |
| **HF DBT (nu)** | 48.05 (32.13 - 65.50) | 53.90 (15.75 - 63.70) | 0.12 |
| **(LF/HF) Rest** | 0.94 (0.64 - 2.15) | 1.21(0.51- 2.46) | 0.90 |
| **(LF/HF) CPT** | 1.03 (0.92 - 2.13) | 1.12 (0.58 - 1.75) | 0.13 |
| **(LF/HF) DBT** | 1.08 (0.52 -1.90) | 0.87 (0.57- 3.66) | 0.23 |

Data are expressed as median with interquartile range; Statistics: 1. Mann-Whitney U test; CPT: Cold pressor test; DBT: Deep breathing test; IQ: interquartile range; ms: milliseconds; LF: Low frequency; HF: High Frequency; n.u.: normalised unit, * - p Value <0.05
