# Supplementary material for: A Cross-Sectional Study on Central Sensitization and Autonomic Changes in Fibromyalgia
Source: Front Neurosci. 2020 Aug 4;14:788. doi: 10.3389/fnins.2020.00788 (PMC7417433; doi:10.3389/fnins.2020.00788)
Supplement: TABLE S1 — Comparison of frequency domain parameters of heart rate variability among fibromyalgia and control group during rest, CPT and DBT. [file Data_Sheet_1.zip › Table S2.docx]

**A Cross-Sectional Study on Central Sensitization and Autonomic Changes in Fibromyalgia**

**Hazra S^1^, Venkataraman S^2^, Handa G^2^, Yadav SL^2^, Wadhwa S^2^, Singh U^2^, Kochhar KP^3^, Deepak KK^3^, Sarkar K^4^**

**Supplementary table 2 Comparison of frequency domain parameters of heart rate variability among fibromyalgia and control group during rest, CPT and DBT by Wilcoxon and Friedman test with Bonferroni priori**

1. **Total power HRV:**

|  | **FM Total Rest** | **Control Total Rest** | **FM Total CPT** | **Control Total CPT** | **FM Total DBT** | **Control**  **Total DBT** |
| --- | --- | --- | --- | --- | --- | --- |
| **FM Total Rest** |  | 1 | 6.63E-05 | 0.00485 | 9.58E-05 | 0.1715 |
| **Control Total Rest** | 1 |  | 0.05583 | 0.02773 | 0.01927 | 1 |
| **FM Total CPT** | 6.63E-05 | 0.05583 |  | 1 | 1 | 0.5625 |
| **Control Total CPT** | 0.00485 | 0.02773 | 1 |  | 1 | 1 |
| **FM Total DBT** | 9.58E-05 | 0.01927 | 1 | 1 |  | 0.3911 |
| **Control Total DBT** | 0.1715 | 1 | 0.5625 | 1 | 0.3911 |  |

1. **LF (ms^2^):**

|  | **FM LF Rest** | **Control LF Rest** | **FM LF CPT** | **Control LF CPT** | **FM LF DBT** | **Control LF DBT** |
| --- | --- | --- | --- | --- | --- | --- |
| **FM LF Rest** |  | 1 | 0.0003168 | 0.08777 | 0.0004653 | 1 |
| **Control LF Rest** | 1 |  | 0.000945 | 0.0001876 | 0.01001 | 1 |
| **FM LF CPT** | 0.0003168 | 0.000945 |  | 1 | 1 | 0.2961 |
| **Control LF CPT** | 0.08777 | 0.0001876 | **1** |  | 1 | 1 |
| **FM LF DBT** | 0.0004653 | 0.01001 | 1 | 1 |  | 1 |
| **Control LF DBT** | 1 | 1 | 0.2961 | 1 | 1 |  |

1. **LF (nu):**

|  | **FM LF Rest** | **Control LF Rest** | **FM LF CPT** | **Control LF CPT** | **FM LF DBT** | **Control LF DBT** |
| --- | --- | --- | --- | --- | --- | --- |
| **FM LF Rest** |  | 1 | 1 | 1 | 1 | 1 |
| **Control LF Rest** | 1 |  | 1 | 0.4991 | 1 | 1 |
| **FM LF CPT** | 1 | 1 |  | 1 | 1 | 1 |
| **Control LF CPT** | 1 | 0.4991 | 1 |  | 1 | 1 |
| **FM LF DBT** | 1 | 1 | 1 | 1 |  | 1 |
| **Control LF DBT** | 1 | 1 | 1 | 1 | 1 |  |

1. **HF (ms^2^):**

|  | **FM HF Rest** | **Control HF Rest** | **FM HF CPT** | **Control HF CPT** | **FM HF DBT** | **Control HF DBT** |
| --- | --- | --- | --- | --- | --- | --- |
| **FM HF Rest** |  | 1 | 0.0003307 | 0.3721 | 0.009309 | 1 |
| **Control HF Rest** | 1 |  | 0.002649 | 0.001559 | 0.05248 | 0.169 |
| **FM HF CPT** | 0.0003307 | 0.002649 |  | 1 | 1 | 0.1077 |
| **Control HF CPT** | 0.3721 | 0.001559 | 1 |  | 1 | 1 |
| **FM HF DBT** | 0.009309 | 0.05248 | 1 | 1 |  | 1 |
| **Control HF DBT** | 1 | 0.169 | 0.1077 | 1 | 1 |  |

1. **HF (nu):**

|  | **FM HF Rest** | **Control HF Rest** | **FM HF CPT** | **Control HF CPT** | **FM HF DBT** | **Control HF DBT** |
| --- | --- | --- | --- | --- | --- | --- |
| **FM HF Rest** |  | 1 | 1 | 1 | 1 | 1 |
| **Control HF Rest** | 1 |  | 1 | 0.3718 | 1 | 1 |
| **FM HF CPT** | 1 | 1 |  | 1 | 1 | 1 |
| **Control HF CPT** | 1 | 0.3718 | 1 |  | 1 | 1 |
| **FM HF DBT** | 1 | 1 | 1 | 1 |  | 1 |
| **Control HF DBT** | 1 | 1 | 1 | 1 | 1 |  |

1. **LF/HF:**

|  | **FM (LF/HF) Rest** | **Control (LF/HF) Rest** | **FM (LF/HF) CPT** | **Control (LF/HF) CPT** | **FM (LF/HF) DBT** |
| --- | --- | --- | --- | --- | --- |
| **FM (LF/HF) Rest** | | 1 | 1 | 1 | 1 |
| **Control (LF/HF) Rest** | 1 |  | 1 | 1 | 1 |
| **FM (LF/HF) CPT** | 1 | 1 |  | 1 | 0.8298 |
| **Control (LF/HF) CPT** | 1 | 1 | 1 |  | 1 |
| **FM (LF/HF) DBT** | 1 | 1 | 0.8298 | 1 |  |
