# Supplementary material for: A Cross-Sectional Study on Central Sensitization and Autonomic Changes in Fibromyalgia
Source: Front Neurosci. 2020 Aug 4;14:788. doi: 10.3389/fnins.2020.00788 (PMC7417433; doi:10.3389/fnins.2020.00788)
Supplement: TABLE S1 — Comparison of frequency domain parameters of heart rate variability among fibromyalgia and control group during rest, CPT and DBT. [file Data_Sheet_1.zip › Table S3.docx]

**A Cross-Sectional Study on Central Sensitization and Autonomic Changes in Fibromyalgia**

**Hazra S^1^, Venkataraman S^2^, Handa G^2^, Yadav SL^2^, Wadhwa S^2^, Singh U^2^, Kochhar KP^3^, Deepak KK^3^, Sarkar K^4^**

**Supplementary table 3 Comparison of peak Oxy-hemoglobin concentration (μM) recorded by fNIRS detectors in the prefrontal cortex during rest and cold pressor test in fibromyalgia patients and control groups.**

| **fNIRS detector site** | **Physiological**  **state** | **FM Group (n=50)** | **Control group (n=50)** | **p Value** |
| --- | --- | --- | --- | --- |
| **1** | **Rest** | 2.90 (2.42 - 3.31) | 2.19 (1.52 - 2.78) | **<0.01*** |
| **1** | **CPT** | 4.28 (3.64 - 5.24) | 4.09 (2.63 - 5.53) | 0.08 |
| **2** | **Rest** | 2.71 (1.93 - 3.27) | 1.59 (0.92 - 2.10) | **<0.01*** |
| **2** | **CPT** | 4.11 (3.67 - 5.18) | 4.13 (1.85 - 6.16) | 0.74 |
| **3** | **Rest** | 2.25 (1.58 - 3.15) | 2.00 (1.05 - 2.84) | 0.53 |
| **3** | **CPT** | 4.29 (3.60 - 5.53) | 2.75 (1.77 - 3.61) | **<0.01*** |
| **4** | **Rest** | 2.37 (1.19 - 3.64) | 1.67 (1.26 - 2.10) | **<0.01*** |
| **4** | **CPT** | 4.04 (3.42 - 5.06) | 3.54 (2.93 - 4.23) | **<0.01*** |
| **5** | **Rest** | 3.03 (1.54 - 4.30) | 1.87(1.47 - 2.10) | **<0.01*** |
| **5** | **CPT** | 5.67 (4.39 - 8.45) | 2.85 (1.36 - 3.41) | **<0.01*** |
| **6** | **Rest** | 3.70 (2.49 - 4.75) | 1.68 (1.03 - 2.53) | **<0.01*** |
| **6** | **CPT** | 5.59 (4.70 - 6.35) | 3.83 (2.64 - 6.02) | **<0.01*** |
| **7** | **Rest** | 2.91 (1.20 - 3.68) | 2.55 (1.23 - 3.26) | 0.43 |
| **7** | **CPT** | 5.10 (4.16 - 9.11) | 3.35 (1.26 - 4.13) | **<0.01*** |
| **8** | **Rest** | 3.34 (1.99 - 4.71) | 1.22 (0.66 - 2.96) | **<0.01*** |
| **8** | **CPT** | 5.06 (4.19 - 6.54) | 4.54 (2.96 - 9.81) | 0.9 |
| **9** | **Rest** | 2.67 (1.24 - 3.32) | 1.99 (1.02 - 3.40) | 0.22 |
| **9** | **CPT** | 5.02 (3.87 - 7.48) | 3.76 (2.52 - 6.16) | **<0.01*** |
| **10** | **Rest** | 2.57 (1.60 - 3.47) | 2.54 (1.62 - 4.65) | 0.38 |
| **10** | **CPT** | 4.66 (3.48 - 5.63) | 4.68 (3.37 - 10.37) | **0.01*** |
| **11** | **Rest** | 3.09 (2.30 - 4.03) | 2.53 (1.73 - 3.21) | **<0.01*** |
| **11** | **CPT** | 4.79 (3.82 - 5.72) | 5.96 (3.92 - 7.49) | **<0.01*** |
| **12** | **Rest** | 2.81 (1.38 - 3.73) | 2.77 (2.00 - 4.96) | **0.04*** |
| **12** | **CPT** | 4.66 (3.46 - 5.68) | 4.39 (1.78 - 7.15) | 0.91 |
| **13** | **Rest** | 3.28 (2.73 - 3.94) | 1.78 (1.17 - 2.77) | **<0.01*** |
| **13** | **CPT** | 5.11 (3.96 - 6.58) | 5.22 (3.97 - 6.90) | 0.61 |
| **14** | **Rest** | 3.38 (2.18 - 5.40) | 1.89 (1.34 - 2.52) | **<0.01*** |
| **14** | **CPT** | 4.95 (3.03 - 6.51) | 4.69 (3.73 - 5.62) | 0.42 |
| **15** | **Rest** | 3.81 (2.87 - 8.38) | 1.76 (1.43 - 2.88) | **<0.01*** |
| **15** | **CPT** | 5.53 (4.47 - 6.78) | 5.24 (4.48 - 8.65) | 0.83 |
| **16** | **Rest** | 2.93 (2.39 - 3.77) | 2.36 (1.93 - 2.78) | **<0.01*** |
| **16** | **CPT** | 5.08 (2.66 - 6.55) | 3.21 (2.79 -11.04) | 0.33 |

Data are expressed as median with interquartile range; Statistics: Mann-Whitney U test; * - p Value <0.05; μM: micro molar, CPT: cold pressor test
